# The coelacanth rostral organ is a unique low-resolution electro-detector that facilitates the feeding strike

**DOI:** 10.1038/srep08962

**Published:** 2015-03-11

**Authors:** Rachel M. Berquist, Vitaly L. Galinsky, Stephen M. Kajiura, Lawrence R. Frank

**Affiliations:** 1 Center for Scientific Computation in Imaging, Department of Radiology, University of California San Diego, La Jolla, California 92037; 2 Department of Electrical and Computer Engineering, University of California San Diego, La Jolla, California 92093; 3 Department of Biological Sciences, Florida Atlantic University, Boca Raton, Florida 33431; 4 VA San Diego Healthcare System, San Diego, California 92161

## Abstract

The cartilaginous and non-neopterygian bony fishes have an electric sense typically comprised of hundreds or thousands of sensory canals distributed in broad clusters over the head. This morphology facilitates neural encoding of local electric field intensity, orientation, and polarity, used for determining the position of nearby prey. The coelacanth rostral organ electric sense, however, is unique in having only three paired sensory canals with distribution restricted to the dorsal snout, raising questions about its function. To address this, we employed magnetic resonance imaging methods to map electrosensory canal morphology in the extant coelacanth, *Latimeria chalumnae*, and a simple dipole ‘rabbit ears' antennae model with toroidal gain function to approximate their directional sensitivity. This identified a unique focal region of electrosensitivity directly in front of the mouth, and is the first evidence of a low-resolution electro-detector that solely facilitates prey ingestion.

Electrosensitive fishes possess the ability to detect and orient to weak, low frequency electric fields produced by living tissues in contact with water. These signals typically indicate the presence and location of potential prey, conspecifics, or predators at close range^1^. This is an ancient sensory modality that arose early in the radiation of the vertebrate clade, purportedly in osteostracid agnathans in the mid-Silurian around 428 MYA^2^. Electrosensitivity subsequently arose independently in ancestors of all aquatic vertebrate lineages, although it was subsequently lost in hagfish, frogs and toads, amniotes, and neopterygian bony fishes, and then re-evolved independently in two teleost lineages^3^, and in mammals, including the monotremes, and at least one species of dolphin^4^.

Chondrichthyans, non-neopterygian bony fishes, and non-amniote sarcopterygians (i.e. elasmobranchs, holocephalans, bichirs, chondrosteans, coelacanths, lungfish and amphibians) possess an electric sense inherited from a common ancestor, with Lorenzinian-type ampullary canals innervated by the dorsal root of the anterior lateral line nerve, and a dorsal octavolateral nucleus in the hindbrain responsible for processing all primary electrosensory inputs^5^. With the exception of coelacanths, the electric sense in this group typically comprises of hundreds, or sometimes thousands, of sensory canals distributed broadly over the dorsal and ventral surface of the head and around the mouth, with these canals arranged in clusters resembling arrays of directional antennae^6^,^7^. This morphology facilitates neural encoding of local electric field intensity, orientation, and polarity^8^, enabling these predators to localize nearby bioelectric field sources at various relative angles of approach^1^,^9^.

Devonian marine coelacanths possessed a rostral organ on their snouts as evidenced from their fossilized chondrocrania^10^. The rostral organs of these lobe-finned fishes exhibited similarities to those of placoderms, with the organ being broadly exposed to the environment^2^. In contrast, more modern coelacanths, including the two species of living marine coelacanth that are the sole survivors of this once diverse order of fishes^11^, possess a rostral organ morphology unique to this group, in which an enclosed rostral organ communicates with the environment via three pairs of discrete ‘tubules', with pores opening on the snout^12^,^13^,^14^. Studies of the anatomy of the rostral organ in the extant species, *Latimeria chalumnae*, as well as its innervation and central nervous system nuclei, found similarities with ampullary electrosensory systems of other marine fishes, leading to the conclusion that the rostral organ is a Lorenzinian-type electroreceptive structure^14^,^15^,^16^. Electrosensory capabilities presumably mediated by the rostral organ were confirmed with anecdotal behavioral observations of *L. chalumnae* biting at induced weak electric field sources^17^.

However, the morphology of the coelacanth electric sense remains unique among present day fishes in having only three pairs of sensory canals, called ‘tubules', all of which are restricted in distribution to a small area of the dorsal snout and with sensory epithelia clustered inside a single central chamber in the ethmoid region^14^. The coelacanth electric sense is also unusual in having no electroreceptors associated with the ventral surface or lower jaws.

Due to their critically endangered status prohibiting access to living coelacanths for research^18^, and also the scarcity of accessible specimens housed in natural history collections, it is probably not surprising that the functional significance of this unique morphology has never been addressed. Fortunately, we had an opportunity to employ non-invasive magnetic resonance imaging (MRI) methods to precisely map the three-dimensional spatial organization of the rostral organ of a preserved West Indian Ocean coelacanth, *Latimeria chalumnae*. We then used these data to implement a simple dipole ‘rabbit ears' antennae model with toroidal gain function to approximate the directional sensitivity of each tubule pair and hence estimate the spatial selectivity of the unique electrosensory system of this group of fishes for the first time.

## Results

**Figure 1:**
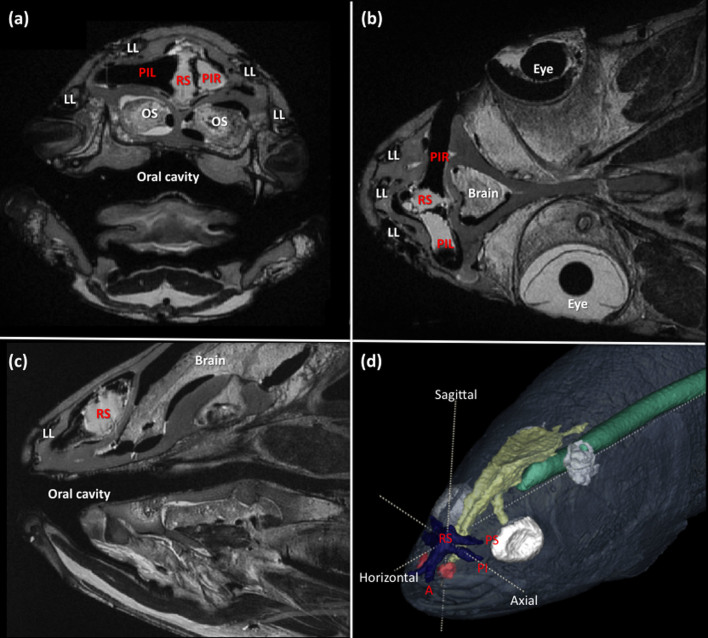
Morphology of the rostral organ electric sense in the extant coelacanth, *L. chalumnae*. MRI grey-scale data showing (a) axial, (b) horizontal, and (c) sagittal plane cross-sectional slices through the head at the location of the rostral organ. (d) 3D reconstruction from image segmentation of MRI grey-scale data showing in situ rostral organ morphology (depicted in blue). A selection of rostral organ structures have been annotated following the terminologies of Bemis & Hetherington^14^ with abbreviations provided below. The rostral sac (RS) resides within the median rostral cavity in the ethmoid portion of the chondrocranium. It contains all of the electrosensory epithelia and comprises a system of crypts that are invaginated into the rostral sac tissues. Three pairs of tubules radiate out from the rostral sac to pores opening on the surface of the snout. These bilaterally paired tubules comprise the anterior (A), posterior inferior (PI), and the posterior superior (PS) tubules. Note that in life, the spaces within the rostral sac and tubule systems are filled with a gelatinous substance that was stripped away as a result of tissue fixation and preservation. The thin layer of fatty tissue that surrounds the tubules and rostral sac in life has similarly been lost. Abbreviations: A, anterior tubule; LL, lateral line canal; OS, olfactory structures; PI, posterior inferior tubule; PIL, left posterior inferior tubule; PIR, right posterior inferior tubule; PS, posterior superior tubule; RS, rostral sac.

The electrosensory anatomy of *Latimeria* *chalumnae* observed in cross-sectional MRI slices (Fig. 1a–c) agreed well with existing anatomical descriptions using dissection material^12^,^13^,^14^. Results of three-dimensional image segmentation however, identified some features of the coelacanth electric sense not previously reported (Fig. 1d). All rostral organ tubules were approximately equivalent in length and volume, indicating similar sensitivities to electric field strength^19^ (Table 1). We also found the range of tubule orientations in the rostral organ array to be relatively restricted when compared with the small amount of data available on other fishes^19^,^20^, with a maximum range in vertical inclination (∝^v^) of only 91.3°, and maximum range in rotation angle in the horizontal plan (∝^h^) of 253° (Table 2).

**Figure 2:**
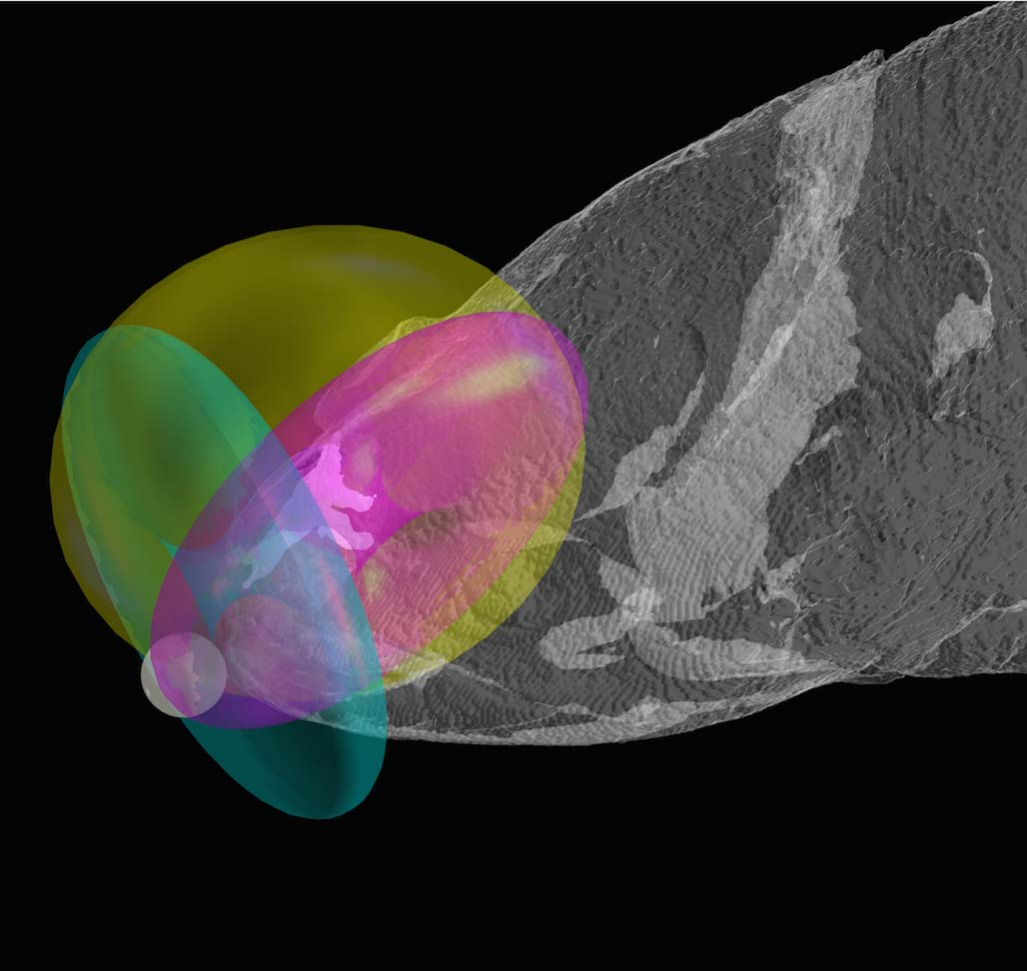
Antennae gain function plots for the three pairs of rostral organ electrosensory tubules in the extant coelacanth, *L. chalumnae*. The rostral organ located in the ethmoid region of the dorsal snout is depicted in white. Each of its three pairs of electrosensory tubules are represented by a simple dipole “rabbit ears” antenna with toroidal gain function (posterior superior pair (yellow), posterior inferior pair (pink), and anterior pair (green)). The region of overlap of these three tori corresponds to the localized electrosensory detection area, which is depicted by the white sphere in close proximity to the front of the mouth.

Electrical antennae gain function plots, estimating the spatial configuration of rostral organ electrosensitivity, produced three discrete overlapping tori surrounding the dorsal and lateral snout in front of the eyes, and one region of overlap situated in a relatively restricted region of space in front of the mouth (Fig. 2). This region of toroidal overlap, depicted by the white sphere, outlines an area in space of approximately equal (or balanced) sensitivity for all of the tubule electrosensors, and is where the rostral organ is expected to have maximum functional sensitivity. Different prey can generate electric fields of different strengths, and thus can be sensed at varying gain levels, depending on the sensory threshold used for the detectors. The exact shape of the overlapping, or balanced, sensory region will therefore vary with changes in gain, from a single point (or a sphere with a very small radius) when the gain is very small, to a larger, more complex shaped area enclosed by surfaces of nested tori when the gain is increased. However, its general location in the vicinity of the mouth does not significantly change, irrespective of sensitivity threshold, or of prey field strength.

In our model, when prey is located close to the detectors at any of the toroidal shaped isosurfaces of the electrosensory array, its presence roughly produces the same electrical potential across the detectors, thus giving maximum gain when it is positioned at right angles to the rabbit ear dipoles. This is the result we would expect if the configuration of the coelacanth rostral organ array could indeed function as an antenna. These results therefore demonstrate that our antenna gain function approach can provide a valid framework for describing how an electric dipole stimulus, such as that generated by prey, can be sensed by a moving electrosensory array, such as that possessed by the coelacanth, *L. chalumnae*.

## Discussion

With only three pairs of electrosensory tubules, the coelacanth electric sense is a low-resolution electro-detector. These fish therefore have limited sensitivity to the directional properties of electric fields, and are probably not capable of extracting complex spatial information for localizing bioelectric field sources at different orientations relative to their head^1^,^8^. This makes them unique among extant electrosensitive fishes in having limited ability to discriminate the relative movements of prey, with their electric sense having little, or perhaps no, involvement in tracking prey. Instead, these fish have a spatially selective electric sense with targeted sensitivity specifically associated with the mouth. To date, the only other electric sense known to comprise of so few electrosensory elements, which are also restricted to the dorsal snout, is that of the Guiana dolphin, *Sotalia* *guianensis*^4^, suggesting its electric sense may have similar functionality to that of coelacanth fishes.

Marine electrosensitive fishes typically possess a diversity of ampullary canal lengths and orientations that is assumed to reflect differential sensitivity to electric field strength and direction, and thus a degree of sensory specialization within their arrays^19^,^20^. The ampullary canals of *Latimeria chalumnae*, however, are all of similar length and volume and thus sensitivity, with relatively limited spatial coverage, suggesting a single functional role for the array. Since our model identified a localized region of electrical sensitivity located directly in the vicinity of the mouth, we hypothesize sensory contributions from the rostral organ are important once prey is already within striking distance, and thus has a function directly related to prey intake.

These findings are intriguing in light of the peculiar locomotory and feeding behaviors reported by H. Fricke and colleagues^17^,^21^,^22^ in the only field observations yet made of *L. chalumnae*. Although capable of high burst speeds and adeptly maneuvering its fleshy fins to accelerate, brake, and turn its body in any direction, including upside-down, this fish is unusual in being a nocturnal drift-hunting predator that does not actively swim in search of food^21^. Instead, it encounters small benthic prey hidden among rocks and crevices as it drifts virtually motionless with the currents above the seafloor, and has been frequently observed doing this in a bizarre ‘head-stand' orientation^22^. Anecdotal evidence of electrosensory involvement in this drift-hunting behavior was obtained with weak electric fields reportedly eliciting these head-stand behaviors^17^. Since they are passive drift-hunters, rather than active foragers which track down prey, we suspect the coelacanth would benefit little from having a broadly directional electrosensory array.

*Latimeria chalumnae* also has a very large terminal mouth with protrusible jaws^23^. The bite is further aided by its lower jaws which have a muscular, expandable ‘gular' structure that increases the power and size of its gape, and an intracranial joint, thought to play a role in increasing the mobility of the jaws by allowing movement of the head relative to the trunk, although this function has recently come into question^24^. This morphology facilitates rapid, deliberate jaw movements to powerfully draw in large volumes of water and bring targeted prey into the mouth, a feeding mechanism called suction-inhalation^25^. While drift hunting, these predators have been observed using this mechanism to engulf prey from an estimated 10–20 cm in front of the mouth, all within less than one second^26^. Since we assume this type of feeding mode is not reliant on detailed sensory mapping for orienting precisely aimed, direct bites, it is possible this behavior occurs as soon as prey is within range of its narrowly focused electric sense. We therefore suspect the coelacanth would benefit little from acquiring highly resolved electrosensory images.

It is interesting that almost all other cartilaginous and non-neopterygian bony fishes possess ventral mouths and have multiple groups of electroreceptors associated with the ventral surface, including around the mouth^27^. However, species with mouths located in more terminal positions (similar to coelacanths), have significantly fewer electroreceptors and canal groupings, particularly associated with the ventral surface and mouth^28^,^29^,^30^. These species are typically filter-feeders^28^,^29^ or sit-and-wait ambush predators^30^, where it is assumed a more highly resolved electrosensory image is less critical for prey capture^27^.

Recent findings from a series of multisensory knock-out experiments in three species of sharks exhibiting different feeding strategies^31^ may shed some light on the possible specialized function of the electric sense in coelacanths. With electroreception blocked, two of the shark species successfully tracked down prey with other intact senses but did not bite at it unless it came into physical contact somewhere near the mouth. The third species could also track down prey but would not consume it even if it touched the mouth, suggesting electrosensory inputs are critical for coordinating jaw movements for feeding in these predators (although it is not known if inputs from the entire sensory array, or a subset of canals, are necessary).

In coelacanths, we hypothesize that the rostral organ is strictly involved in coordinating appropriate feeding responses during the final stage of predation, i.e. the prey strike, and has no broader function in actively tracking and localizing prey to within reach of the mouth, in contrast to other fishes. For example, when motivated to hunt, electrical sources coming in range of this narrowly focused electro-detector may alert the coelacanth to the presence of nearby prey and immediately trigger jaw movements associated with suction-inhalation feeding. It is likely this feeding strategy is most efficient when the predator is foraging perpendicular to the substrate in a head-stand position. This not only increases the likelihood of its benthic prey passing directly in front of the oral cavity (as opposed to below the head if it were drifting parallel to the substrate), but also significantly increases the strike forces available for drawing prey off the substrate, since these forces are aimed primarily downward in that direction^32^.

Although coelacanths, and possibly also the Guiana dolphin^4^, possess the most spatially focused, low resolution electrosensory arrays known, we expect the possession of electrosensory canal groupings focused on the region of space directly within reach of the mouth, may be common to all vertebrate electrosensory arrays. For instance, the small isolated clusterings of mandibular ampullary canals associated with the lower jaws of chondrichthyan fishes^6^,^19^,^20^,^30^,^33^ might serve this function. These groups of electroreceptors may help optimize the efficiency of aquatic predatory strikes by informing the predator precisely when to open its mouth to draw in prey. Other groups of electroreceptors may be more broadly focused on orienting the mouth to within reach of prey, particularly if other sensory inputs are obscured, for example, by substrate, turbulence, or nocturnal conditions^31^. Positioning the mouth within reach of prey, as well as precisely timing the bite to enhance prey capture, are both fundamental, though different, aspects of successful prey capture.

It is interesting to consider the evolution of the vertebrate electric sense, and its subsequent loss and re-evolution, in light of how these aquatic organisms have optimized the efficiency of their predatory strike. Recent discoveries of rich fossil beds with relatively complete fish specimens, some even with traces of soft tissues^34^, are also poised to provide new information on morphological and behavioral innovations that may have been occurring at the time of electrosensory diversifications, and losses. For example, mapping changes in development of peripheral electrosensory structures, changes in the anatomy, size and position of oral structures, changes in the relative position of the eyes and mouth, and changes in locomotory capabilities of various groups may provide much needed insight.

## Methods

### Reconstructing rostral organ morphology

A preserved 950 mm standard length adult male West Indian Ocean coelacanth, *Latimeria chalumnae*, Smith 1939 (Institutional ID: SIO 75-347), was obtained from the Marine Vertebrate Collection (MVC) at the Scripps Institution of Oceanography, UCSD. Use of this specimen was carried out in accordance with approved institutional guidelines for the acquisition of natural history specimens. It was imaged at the Keck Center for Functional Magnetic Resonance Imaging (CFMRI) at UCSD on a 3T (127.7 MHz) human clinical scanner (Signa Excite 750, GE Healthcare, Milwaukee, WI) equipped with a 55 cm bore with full 45 cm field-of-view (FOV) imaging capability and maximum gradient strengths 4.4 mT/m (across the bore), with a maximum slew rate of 250 mT/m/ms and a rise time of 150 ms. A GE 8-channel human head coil (MRI Devices, Waukesha, WI) was used to acquire T2-weighted images with a 3D Cube acquisition pulse sequence in the axial plane at 290 mm^3^ voxel resolution with the following parameters: 90° flip angle, 2500 ms repetition time, 94.77 ms echo time, 122 kHz bandwidth, 150 × 150 × 128 mm in-field field-of-view and 2 averages. Image data were converted to DICOM (http://medical.nema.org/) format for image processing and visualization. The gross morphology of the rostral organ, including the individual pores, tubules and central rostral sac, as well as the skin, brain, eyes, nasal cavity, and a handful of other readily visible structures, were reconstructed using both semi-automatic segmentation and manual segmentation tools in ITK-SNAP (Insight Segmentation and Registration Toolkit; http://www.itksnap.org/). The volume of the rostral organ and its constituent tubule components were calculated from segmented grey-scale MRI data using the ITK-SNAP Volume and Statistics toolbox. The relative angles (α) between each rostral organ tubule with respect to vertical (α*^v^*) and horizontal (α*^h^*) body axes were also calculated, as follows:
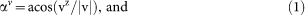

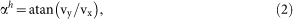
where v_x_, v_y_, and v_z_ are directional cosines of the principal axis of the tubule.

### Estimating rostral organ spatial selectivity

Fjallbrant et al.^35^ previously introduced the concept of an electrical antenna when describing the functioning of the electrosensory system in platypus. Here, we implemented a novel dipole ‘rabbit ears' antennae model with toroidal gain function as a method of approximating the spatial selectivity of the electrosensory rostral organ in *Latimeria chalumae*. The range of toroidal antenna gain we selected was informed by field behavioral observations reported in Fricke and Hissman^26^, who recorded feeding strike initiations in *L. chalumnae* that were initiated when prey was approximately 10–20 cm in front of the snout. The isosurfaces of the gain function we use for the simple dipole antenna are shaped like a series of embedded toroids (doughnuts) symmetrical about the axis of the dipole. The sensitivity of each individual electrosensory rostral organ tubule reaches a maximum when the electric potential gradient (or the electric field) is aligned along it, i.e. along the dipole axis of the ‘rabbit ears' antennae, dropping off to zero as it approaches 90°, following a cosine function^9^.

For our model, the spherical wave decomposition (SWD) methods of Galinsky and Frank^36^ were employed to compute the principal axis of each segmented rostral organ tubule using their smoothed SWD volume for *L* = *N* = 5 or 7. The smoothed SWD volume was then used to construct the inertia tensor as
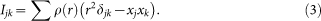


The singular value decomposition was used to diagonalize the inertia tensor *I_jk_* and find the principal axes of the volumes. This was roughly located in between the rostral organ tubules. To localize an area in the surrounding space where all three antennae are estimated to have approximately the same gain, we plotted a single toroidal isosurface for each tubule with the same parameters for each ring torus, i.e. the same radius of an internal center area (a ‘hole') and the same radius of an external ring. Although we did not know the exact value for the gain to use at the surface of each tori, with this simple geometrical construction we do know that the overlapping area that is generated represents the balanced maximum sensitivity for all three antennas.

## Author Contributions

R.M.B. was involved in all aspects of the project. L.R.F. conducted the magnetic resonance imaging. V.L.G. generated the antennae gain function model and 3D rendering. S.M.K. provided expertise on electrosensory morphology and the interpretation of results. All authors contributed to data analysis and manuscript preparation.

## Figures and Tables

**Table 1:** Comparison of rostral organ structure dimensions

Rostral Organ Structure	Volume (mm^3^)	Mean Volume ± SD (mm^3^)	Length (mm)	Mean Length ± SD (mm)
Anterior tubule (L)	1370	1353.5 ± 23.3	34.38	35.16 ± 1.10
Anterior tubule (R)	1337		35.94	
Posterior Inferior tubule (L)	1405	1368 ± 52.3	39.15	38.29 ± 1.22
Posterior Inferior tubule (R)	1331		37.43	
Posterior Superior tubule (L)	1629	1639.5 ± 14.8	31.24	32.19 ± 1.34
Posterior Superior tubule (R)	1650		33.13	
Medial cavity	1810	---	---	---
Full Rostral Organ	10532	---	---	---

L = left; R = right.

**Table 2:** Rostral organ tubule orientations

Rostral Organ Tubule	Inclination Angle Relative to Head-Tail Axis (α^v^)	Rotation Angle in Plane Orthogonal to Head-Tail Axis (α^h^)
Anterior (L)	40.05°	−100.68°
Anterior (R)	41.42°	−100.29°
Posterior Inferior (L)	113.77°	152.83°
Posterior Inferior (R)	125.81°	−55.88°
Posterior Superior (L)	111.88°	10.26°
Posterior Superior (R)	132.75°	−2.63°

L = left; R = right.
